# Diversity of Microorganisms in Biocrusts Surrounding Highly Saline Potash Tailing Piles in Germany

**DOI:** 10.3390/microorganisms9040714

**Published:** 2021-03-30

**Authors:** Ekaterina Pushkareva, Veronika Sommer, Israel Barrantes, Ulf Karsten

**Affiliations:** 1 Department of Applied Ecology and Phycology, Institute of Biological Sciences, University of Rostock, 18059 Rostock, Germany; veronika.sommer@uni-rostock.de (V.S.); ulf.karsten@uni-rostock.de (U.K.); 2 Department of Biology, Botanical Institute, University of Cologne, 50674 Cologne, Germany; 3 Research Group Translational Bioinformatics, Institute for Biostatistics and Informatics in Medicine and Ageing Research, Rostock University Medical Center, 18057 Rostock, Germany; israel.barrantes@uni-rostock.de

**Keywords:** biocrusts, diversity, microorganisms, potash mining, high-throughput sequencing

## Abstract

Potash tailing piles located in Germany represent extremely hypersaline locations that negatively affect neighbouring environments and limit the development of higher vegetation. However, biocrusts, as cryptogamic covers, inhabit some of these areas and provide essential ecological functions, but, nevertheless, they remain poorly described. Here, we applied high-throughput sequencing (HTS) and targeted four groups of microorganisms: bacteria, cyanobacteria, fungi and other eukaryotes. The sequencing of the 16S rRNA gene revealed the dominance of Proteobacteria, Cyanobacteria and Actinobacteria. Additionally, we applied yanobacteria-specific primers for a detailed assessment of the cyanobacterial community, which was dominated by members of the filamentous orders Synechococcales and Oscillatoriales. Furthermore, the majority of reads in the studied biocrusts obtained by sequencing of the 18S rRNA gene belonged to eukaryotic microalgae. In addition, sequencing of the internal rDNA transcribed spacer region (ITS) showed the dominance of Ascomycota within the fungal community. Overall, these molecular data provided the first detailed overview of microorganisms associated with biocrusts inhabiting highly saline potash tailing piles and showed the dissimilarities in microbial diversity among the samples.

## 1. Introduction

The production of potash fertilizers results in great amounts of salt waste, which is deposited as potash tailing piles. Potash mainly consists of the by-product NaCl and, therefore, has negative effects on the environment. The impact of salt from the potash tailing piles is reflected in the surrounding ecosystems, which leads to anthropogenic salinization of ground water, freshwater systems and soil [1]. Due to the limited higher plant vegetation in the surroundings of the potash tailing piles, biocrusts appear to play an important role. They consist of several micro-(microalgae, cyanobacteria, bacteria, microfungi) as well as macro-organisms (lichens, mosses) that live in the first millimetres of the soil [2]. Biocrusts often prevail in extreme environments where higher plant establishment is inhibited due to harsh abiotic conditions.

Considering that without a base sealing, large amounts of NaCl from the potash tailing piles leak into the surroundings (soil, ground water etc.), halophytes common in coastal habitats [3,4], as well as salt-tolerant fauna [5,6], are getting established in these anthropogenically disturbed areas. Various studies have addressed the occurrence of microbial phototrophs [7], fungi [8,9,10] and bacteria as well as Archaea [11,12,13,14] in hypersaline environments alone. However, microbial community composition in biocrusts from naturally saline habitats was described in a few studies only [15,16] and that of German potash tailing piles exclusively by Sommer et al. [17,18]. Besides, both latter studies, using traditional culture-dependent methods, showed a diverse community of microbial phototrophs in the biocrusts [17,18].

There are many potash tailing piles in Germany. Some of them are still active and continue to enlarge because of fertilizer mining. Therefore, it is crucial to understand the impact of salt on biocrust microbiota inhabiting the surroundings. Here, we applied high-throughput sequencing (HTS) to obtain thorough and comprehensive insight into biocrust microbial community composition, with an emphasis on bacteria, cyanobacteria, fungi and other eukaryotes in the previously partially described biocrusts [17,18]. These data will help us to better understand the presence and abundance of specific salt-tolerant microorganisms in anthropogenically disturbed mining areas. In addition, such information is essential for any restoration efforts of potash tailing piles by, for example, artificial establishment of biocrusts in order to lower the salt leachate.

## 2. Materials and Methods

A total of twelve highly saline biocrust samples from the areas surrounding five potash tailings piles in Germany (Shreyahn (SY), Oedesse (OD), Wietze (WT), Teutschenthal (TT), the not named site (NN)) were used in this study. Site descriptions and sampling as well as the chemical parameters and microphototrophic community composition were presented in two previous publications [17,18] and are summarised in Supplementary Table S1.

Total DNA was extracted from 250 mg biocrust samples using a PowerSoil DNA Isolation Kit (MOBIO, Carlsbad, CA, USA) according to the manufacturer’s instructions and DNA concentrations were quantified using a Qubit 3.0 Fluorometer. Furthermore, the DNA was sent to Microsynth AG (Balgach, Switzerland), where PCR and sequencing using the Illumina MiSeq platform (v3, 2 × 300 bp) were performed. Four groups of microorganisms were targeted in the amplification step: bacteria (V3–V4 region of the 16S rRNA gene), cyanobacteria (V4 region of the 16S rRNA gene), fungi (rDNA internal transcribed spacer region (ITS)) and other eukaryotes (18S rRNA gene). The primers used in this study and their descriptions are presented in Supplementary Table S2. The raw sequencing data were deposited in the European Nucleotide Archive (ENA) under the accession number PRJEB43759.

Quality control and assembly of the reads were performed using the PANDAseq program (version 2.11 [19]). Operational taxonomic units (OTUs) were obtained using USEARCH (version 6.1.544 [20]) from the *pick_open_reference_otus.py* script of QIIME 1.9.1 [21] and later assigned to the appropriate taxa using the following databases with 97% identity threshold: 16S rRNA Greengenes (version 13.8 [22]) for the bacteria and cyanobacteria; UNITE (version 12_11 [23]) for the fungi and SILVA (version 132 [24]) for the other eukaryotes. Several samples resulted in the absence or a low number of reads and thus were excluded from any further analyses (Supplementary Table S1). Eventually, the highly saline conditions of biocrusts seemed to interfere with DNA extraction or even further with PCR, as it was challenging to isolate pure algal cultures from the studied samples in the previous study [18].

The statistical analyses and figures were performed in RStudio (version 1.4.1106). Alpha diversity indices (Shannon and Simpson) were calculated using the package *vegan* [25]. The community dissimilarities between the studied biocrust samples were evaluated using a similarity profile routine (SIMPROF [26]). Pearson correlation coefficients were calculated to assess the relationship between alpha diversity indices and soil parameters such as pH, total organic carbon (TOC), total organic nitrogen (TON) and the C/N ratio.

## 3. Results and Discussion

High-throughput sequencing produced 143,936 bacterial, 22,655 cyanobacterial, 407,178 eukaryotic and 155,349 fungal reads (1527, 87, 329 and 189 OTUs, respectively). These numbers were much lower than those observed in other biocrusts [27,28,29]. The low number of reads, together with several failed samples, could suggest that highly saline conditions might either inhibit biocrust microbiota or negatively influence DNA extraction.

**Figure 1:**
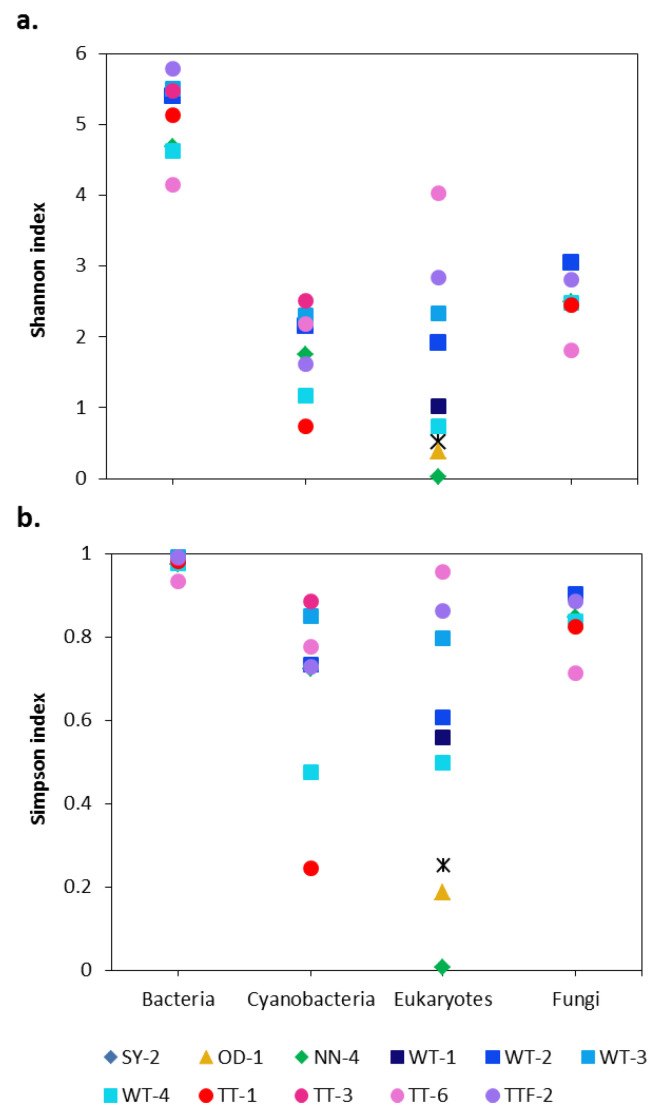
Alpha diversity indices in the studied biocrusts: (**a**) Shannon and (**b**) Simpson. SY, OD, WT, TT and NN correspond to Shreyahn, Oedesse, Wietze, Teutschenthal and the not named site, respectively.

**Figure 2:**
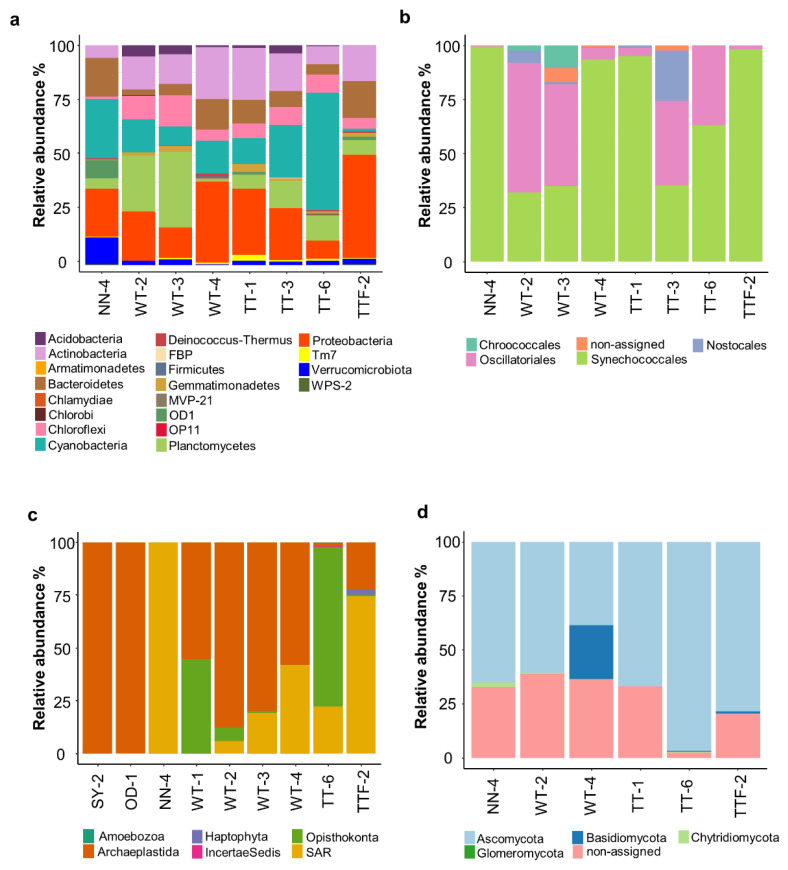
Relative abundance of bacteria (**a**), cyanobacteria (**b**), eukaryotes (**c**) and fungi (**d**) in the studied biocrusts. SY, OD, WT, TT and NN correspond to Shreyahn, Oedesse, Wietze, Teutschenthal and the not named site, respectively.

The alpha diversity indices revealed higher bacterial diversity across the studied biocrusts than other groups of microorganisms (Figure 1). In general, the bacterial community in the biocrusts surrounding potash tailing piles was dominated by Proteobacteria (29% of total bacterial reads, 446 OTUs), Cyanobacteria (16% of total bacterial reads, 87 OTUs), Actinobacteria (15% of total bacterial reads, 255 OTUs), Planctomycetes (14% of total bacterial reads, 227 OTUs), Bacteroidetes (10% of total bacterial reads, 181 OTUs) and Chloroflexi (8% of total bacterial reads, 124 OTUs) (Figure 2a). Overall, the distribution of bacterial orders in the studied biocrusts was similar to those of other biocrusts around the world [27,30,31,32]. Besides, representatives of Bacteroidetes and Gammaproteobacteria were shown to be salt-tolerant in soils [33]. In addition, genera such as *Marinobacter* (19 OTUs), *Halomonas* (three OTUs) and *Halorhodospira* (one OTU) identified within the Gammaproteobacteria as well as the genus *Salinimicrobium* (two OTUs) from the Bacterioidetes are common for saline to extremely saline environments [34,35,36]. However, records of these genera in biocrusts are very rare, which underlines the unique conditions of the studied sampling sites.

The high numbers of cyanobacteria obtained with general bacterial primers in the studied biocrusts confirmed their importance in such extreme environments (Figure 2a). Moreover, a detailed investigation of cyanobacteria with cyanobacteria-specific primers (Figure 2b) showed the dominance of the filamentous orders Synechococcales (64% of total cyanobacterial reads, 54 OTUs) and Oscillatoriales (31% of total cyanobacterial reads, 21 OTUs). The dominance of filamentous cyanobacteria from these two orders has been previously observed in biocrusts from other extreme environments such as, for example, Arctic soils [37,38,39]. In addition, one OTU recorded at two sites (NN and TT) was identified as the salt-tolerant genus *Halomicronema* from the order Synecchococcales [40].

In contrast to the filamentous cyanobacteria, members of the heterocystous order Nostocales, which are able to fix atmospheric nitrogen (N_2_), exhibited much lower abundance (3% of total cyanobacterial reads, six OTUs) than in biocrusts from other parts of Germany [37]. This could indicate that the biocrusts in potash tailing piles are poorly-developed and that ecological processes induced by these N-fixing microorganisms have slowed down [41]. Likewise, members of the order Chroococcales usually constitute a minor fraction within biocrust cyanobacteria [28,37] and only a small proportion of this order was detected in the biocrusts surrounding potash tailing piles (1% of total cyanobacterial reads, three OTUs).

The eukaryotic community in the studied biocrusts was dominated by the clade Archaeplastida (59% of total eukaryotic reads, 95 OTUs; Figure 2c). The phylum Chlorophyta, with the dominance of the two families (Chlorophyceae and Trebouxiophyceae), was the only representative of the clade. These eukaryotic microalgae are typically found in biocrusts [42] and were also reported in the studied potash tailing piles [17,18]. They produce organic osmolytes, which serve as salt protectants [43], and extracellular polymeric substances (EPS) that provide several functions including drought tolerance [44], which, in turn, might have a salt-buffering effect. In addition, the abundance of Archaeplastida increased corresponding to the age of the potash piles. For example, this clade in the two old potash piles (OD and SY) constituted 99.9% of whole eukaryotic community, while in the young piles (NN and TT), it was only 43.5%. Furthermore, the majority of the SAR (Stramenopila, Alveolata and Rhizaria) clade (28% of total eukaryotic reads, 134 OTUs) belonged to the phylum Ochrophyta, which includes diatoms, yellow-green and golden algae. Besides, 17% of total eukaryotic reads corresponded to one OTU assigned to the class Xanthophyceae, which resembled the low number of morphologically identified Xanthophyceae in biocrusts from the potash tailing piles’ surroundings [17].

Sequencing of the ITS region (Figure 2d) showed that Ascomycota was the prevalent fungal phylum, constituting 78% of the total fungal reads (106 OTUs). These fungi, similar to other halotolerant microorganisms, produce exopolysaccharides [45], which might explain their dominance in the highly saline environment. Likewise, Basidiomycota is a potentially halotolerant fungal phylum [45] and a high number of reads was recorded in one sampling site (WT-4). In addition, a large part of the OTUs (21% of total fungal reads) was not assigned to any fungal phyla, either due to database incompleteness or to the presence of new undescribed species.

Multivariate analysis such as SIMPROF based on the amplicon dataset showed that the biocrust samples clustered irrespectively of the origin site, indicating the heterogeneity of microorganisms within individual saline potash tailing piles (Supplementary Figure S1). In addition, soil chemical parameters such as pH, C/N ratio and contents of TOC and TON are well known to support biocrust microbiota [2]. Here, we found no significant correlations between alpha diversity indices and these soil chemical parameters [17], suggesting that other abiotic factors might shape microbial communities in highly saline biocrusts.

## 4. Conclusions

To the best of our knowledge, this study is the first in-depth overview of the microbiota inhabiting highly saline potash tailings piles located in Germany using HTS. Furthermore, this work is complementary to the previous studies in this area and could be used as a baseline for future research aiming to restore potash tailings piles with biocrusts as primary microbiotic vegetation.

## Data Availability

The raw sequencing data are available at the European Nucleotide Archive (ENA) under the accession number PRJEB43759.

## Supplementary Materials

The following are available online at https://www.mdpi.com/article/10.3390/microorganisms9040714/s1. Figure S1: Microbial community dissimilarities between the studied biocrust samples using SIMPROF analysis: a—bacteria, b —cyanobacteria, c—eukaryotes, d—fungi. SY, OD, WT, TT and NN correspond to Shreyahn, Oedesse, Wietze, Teutschenthal and not named site, respectively. Table S1: Biocrust samples collected from in the surroundings of German potash tailings and their description. Table S2: List of primers used in this study and their description.

## References

[1] Cañedo-Argüelles M., Kefford B.J., Piscart C., Prat N., Schäfer R.B., Schulz C.J. (2013). Salinisation of rivers: An urgent ecological issue. Environ. Pollut..

[2] Belnap J., Weber B., Büdel B. (2016). Biological Soil Crusts as an Organizing Principle in Drylands.

[3] Garve E., Garve V. (2000). Halophyten an Kalihalden in Deutschland und Frankreich (Eisass). Tuexenia.

[4] John H. (2000). Zur Ausbreitung von Halophyten und salztoleranten Pflanzen in der Umgebung von Kali-Rückstandshalden am Beispiel des FND “Salzstelle bei Teutschenthal-Bahnhof” (Saalkreis). Mitt. Florist. Kart. Sachsen-Anhalt.

[5] Flügel H.-J., Brenner U. (2013). Die Käferfauna der Salzstellen bei Heringen (Insecta: Coleoptera), ein 50-jähriges Gemeinschaftsprojekt der Arbeitsgemeinschaft Hessischer Koleopterologen. Mitt. Internat. Entomol. Ver. Frankfurt A. M..

[6] Trost M. (2006). Die Historische und Aktuelle Bestandssituation der Halobionten und Halophilen Laufkäferfauna (Coleoptera, Carabidae) im Gebiet der Mansfelder Seen Westlich von Halle/Saale (Sachsen-Anhalt). Hercynia N. F..

[7] Canfora L., Vendramin E., Antisari L.V., Lo Papa G., Dazzi C., Benedetti A., Iavazzo P., Adamo P., Jungblut A.D., Pinzari F. (2016). Compartmentalization of gypsum and halite associated with cyanobacteria in saline soil crusts. FEMS Microbiol. Ecol..

[8] Cantrell S.A., Casillas-Martínez L., Molina M. (2006). Characterization of fungi from hypersaline environments of solar salterns using morphological and molecular techniques. Mycol. Res..

[9] Evans S., Hansen R.W., Stone H.M., Schneegurt M.A. (2013). Isolation and characterization of halotolerant soil fungi from the great salt plains of Oklahoma (USA). Cryptogam. Mycol..

[10] Steiman R., Ford L., Ducros V., Lafond J.L., Guiraud P. (2004). First survey of fungi in hypersaline soil and water of Mono Lake area (California). Antonie Van Leeuwenhoek.

[11] Bachran M., Kluge S., Lopez-Fernandez M., Cherkouk A. (2019). Microbial Diversity in an Arid, Naturally Saline Environment. Microb. Ecol..

[12] Eilmus S., Rösch C., Bothe H. (2007). Prokaryotic life in a potash-polluted marsh with emphasis on N-metabolizing microorganisms. Environ. Pollut..

[13] Fourçans A., De Oteyza T.G., Wieland A., Solé A., Diestra E., Van Bleijswijk J., Grimalt J.O., Kühl M., Esteve I., Muyzer G. (2004). Characterization of functional bacterial groups in a hypersaline microbial mat community (Salins-de-Giraud, Camargue, France). FEMS Microbiol. Ecol..

[14] Oren A. (2008). Microbial life at high salt concentrations: Phylogenetic and metabolic diversity. Saline Syst..

[15] Mikhailyuk T., Vinogradova O.M., Glaser K., Demchenko E.M., Karsten U. (2018). Diversity of terrestrial algae of cape Kazantip (the sea of azov, Ukraine) and some remarks on their phylogeny and ecology. Int. J. Algae.

[16] Pajares S., Escalante A.E., Noguez A.M., García-Oliva F., Martínez-Piedragil C., Cram S.S., Eguiarte L.E., Souza V. (2016). Spatial heterogeneity of physicochemical properties explains differences in microbial composition in arid soils from Cuatro Cienegas, Mexico. PeerJ.

[17] Sommer V., Karsten U., Glaser K. (2020). Halophilic algal communities in biological soil crusts isolated from potash tailings pile areas. Front. Ecol. Evol..

[18] Sommer V., Mikhailyuk T., Glaser K., Karsten U. (2020). Uncovering Unique Green Algae and Cyanobacteria Isolated from Biocrusts in Highly Saline Potash Tailing Pile Habitats, Using an Integrative Approach. Microorganisms.

[19] Masella A.P., Bartram A.K., Truszkowski J.M., Brown D.G., Neufeld J.D. (2012). PANDAseq: Paired-end assembler for illumina sequences. BMC Bioinform..

[20] Edgar R.C. (2010). Search and clustering orders of magnitude faster than BLAST. Bioinformatics.

[21] Caporaso J.G., Kuczynski J., Stombaugh J., Bittinger K., Bushman F.D., Costello E.K., Fierer N., Peña A.G., Goodrich J.K., Gordon J.I. (2010). correspondence QIIME allows analysis of high-throughput community sequencing data Intensity normalization improves color calling in SOLiD sequencing. Nat. Publ. Gr..

[22] McDonald D., Price M.N., Goodrich J., Nawrocki E.P., DeSantis T.Z., Probst A., Andersen G.L., Knight R., Hugenholtz P. (2012). An improved Greengenes taxonomy with explicit ranks for ecological and evolutionary analyses of bacteria and archaea. ISME J..

[23] Abarenkov K., Nilsson R.H., Larsson K.H., Alexander I.J., Eberhardt U., Erland S., Høiland K., Kjøller R., Larsson E., Pennanen T. (2010). The UNITE database for molecular identification of fungi—Recent updates and future perspectives. New Phytol..

[24] Quast C., Pruesse E., Yilmaz P., Gerken J., Schweer T., Yarza P., Peplies J., Glöckner F.O. (2013). The SILVA ribosomal RNA gene database project: Improved data processing and web-based tools. Nucleic Acids Res..

[25] Oksanen J. (2013). Multivariate analysis of ecological communities in R:vegan tutorial. R Doc..

[26] Clarke K., Somerfield P., Gorley R. (2008). Testing of null hypotheses in exploratory community analyses: Similarity profiles and biota-environment linkage. J. Exp. Mar. Bio. Ecol..

[27] Rippin M., Lange S., Sausen N., Becker B. (2018). Biodiversity of biological soil crusts from the Polar Regions revealed by metabarcoding. FEMS Microbiol. Ecol..

[28] Pushkareva E., Pessi I.S., Wilmotte A., Elster J. (2015). Cyanobacterial community composition in Arctic soil crusts at different stages of development. FEMS Microbiol. Ecol..

[29] Pombubpa N., Pietrasiak N., De Ley P., Stajich J.E. (2020). Insights into dryland biocrust microbiome: Geography, soil depth and crust type affect biocrust microbial communities and networks in Mojave Desert, USA. FEMS Microbiol. Ecol..

[30] Zhang B., Kong W., Nan W., Zhang Y. (2016). Bacterial diversity and community along the succession of biological soil crusts in the Gurbantunggut Desert, Northern China. J. Basic Microbiol..

[31] Weber B., Tamm A., Maier S., Rodríguez-Caballero E. (2018). Biological soil crusts of the Succulent Karoo: A review. Afr. J. Range Forage Sci..

[32] Chilton A.M., Neilan B.A., Eldridge D.J. (2018). Biocrust morphology is linked to marked differences in microbial community composition. Plant Soil.

[33] Rath K.M., Fierer N., Murphy D.V., Rousk J. (2019). Linking bacterial community composition to soil salinity along environmental gradients. ISME J..

[34] Ma B., Gong J. (2013). A meta-analysis of the publicly available bacterial and archaeal sequence diversity in saline soils. World J. Microbiol. Biotechnol..

[35] Imhoff J.F. (2001). True marine and halophilic anoxygenic phototrophic bacteria. Arch. Microbiol..

[36] Grimaud R. (2010). Marinobacter. Handbook of Hydrocarbon and Lipid Microbiology.

[37] Williams L., Loewen-Schneider K., Maier S., Büdel B. (2016). Cyanobacterial diversity of western European biological soil crusts along a latitudinal gradient. FEMS Microbiol. Ecol..

[38] Pessi I.S., Pushkareva E., Lara Y., Borderie F., Wilmotte A., Elster J. (2019). Marked Succession of Cyanobacterial Communities Following Glacier Retreat in the High Arctic. Microb. Ecol..

[39] Pushkareva E., Pessi I.S., Namsaraev Z., Mano M.J., Elster J., Wilmotte A. (2018). Cyanobacteria inhabiting biological soil crusts of a polar desert: Sør Rondane Mountains, Antarctica. Syst. Appl. Microbiol..

[40] Abed R.M.M., Garcia-Pichel F., Hernández-Mariné M. (2002). Polyphasic characterization of benthic, moderately halophilic, moderately thermophilic cyanobacteria with very thin trichomes and the proposal of Halomicronema excentricum gen. nov., sp. nov. Arch. Microbiol..

[41] Housman D.C., Powers H.H., Collins A.D., Belnap J. (2006). Carbon and nitrogen fixation differ between successional stages of biological soil crusts in the Colorado Plateau and Chihuahuan Desert. J. Arid Environ..

[42] Büdel B., Dulic T., Darienko T., Rybalka N., Friedl T. (2016). Cyanobacteria and Algae of Biological Soil Crusts. Biological Soil Crusts: An Organizing Principle in Drylands.

[43] Gustavs L., Eggert A., Michalik D., Karsten U. (2010). Physiological and biochemical responses of green microalgae from different habitats to osmotic and matric stress. Protoplasma.

[44] Rossi F., Mugnai G., De Philippis R. (2018). Complex role of the polymeric matrix in biological soil crusts. Plant Soil.

[45] Osińska-Jaroszuk M., Jarosz-Wilkołazka A., Jaroszuk-Ściseł J., Szałapata K., Nowak A., Jaszek M., Ozimek E., Majewska M. (2015). Extracellular polysaccharides from Ascomycota and Basidiomycota: Production conditions, biochemical characteristics, and biological properties. World J. Microbiol. Biotechnol..

