# Supplementary material for: Diversity of Microorganisms in Biocrusts Surrounding Highly Saline Potash Tailing Piles in Germany
Source: Microorganisms. 2021 Mar 30;9(4):714. doi: 10.3390/microorganisms9040714 (PMC8066527; doi:10.3390/microorganisms9040714)
Supplement: Supplementary file 1 [file microorganisms-09-00714-s001.pdf]

Table S1. Biocrust samples collected in the surroundings of German potash tailings and their description.

Sample	Site	Type	pH	C/N	Amplicon Sequencing *			
					Bacteria	Cyanobactertia	Eukaryotes	Fungi
**SY-2**	Shreyahn	young	8.3	21.3			+	
**OD-1**	Oedesse	mature	8.3	35.9			+	
**NN-4**	Not named	mature	7.2	6.9	+	+	+	+
**WT-1**	Wietze	mature	8.1	12.9			+	
**WT-2**		mature	7.9	25.4	+	+	+	+
**WT-3**		mature	8.1	15.7	+	+	+	
**WT-4**		young	8.4	33.9	+	+	+	+
**TT-1**	Teutschenthal	mature	8.5	3.3	+	+		+
**TT-3**		mature	8.8	7.7	+	+		
**TT-6**		mature	8.7	7.6	+	+	+	+
**TTF-2**		young	6.1	13.1	+	+	+	+

*Samples which produced sufficient results by amplicon sequencing marked as "+".

Table S2. List of primers used in this study and their description.

Gene	Organism	Primers	Sequence	Primer Reference
16S	Bacteria	341F	CCTACGGGRSGCAGCAG	[1]
		802R	TACNVGGGTATCTAATCC	
	Cyanobacteria	CYA_F	GGGGAATYTTCCGCAATGGG	
		CYA_R	GACTACWGGGGTATCTAATCCCWTT	
ITS	Fungi	Fungi_F	CAHCGATGAAGAACGYRG	[2]
		Fungi_R	TCCTSCGCTTATTGATATGC	
18S	Eukaryotes	tarEuk_F	CCAGCASCYGCGGTAATTCC	[3]
		tarEUk_R	ACTTTCGTTCTTGATYRA	

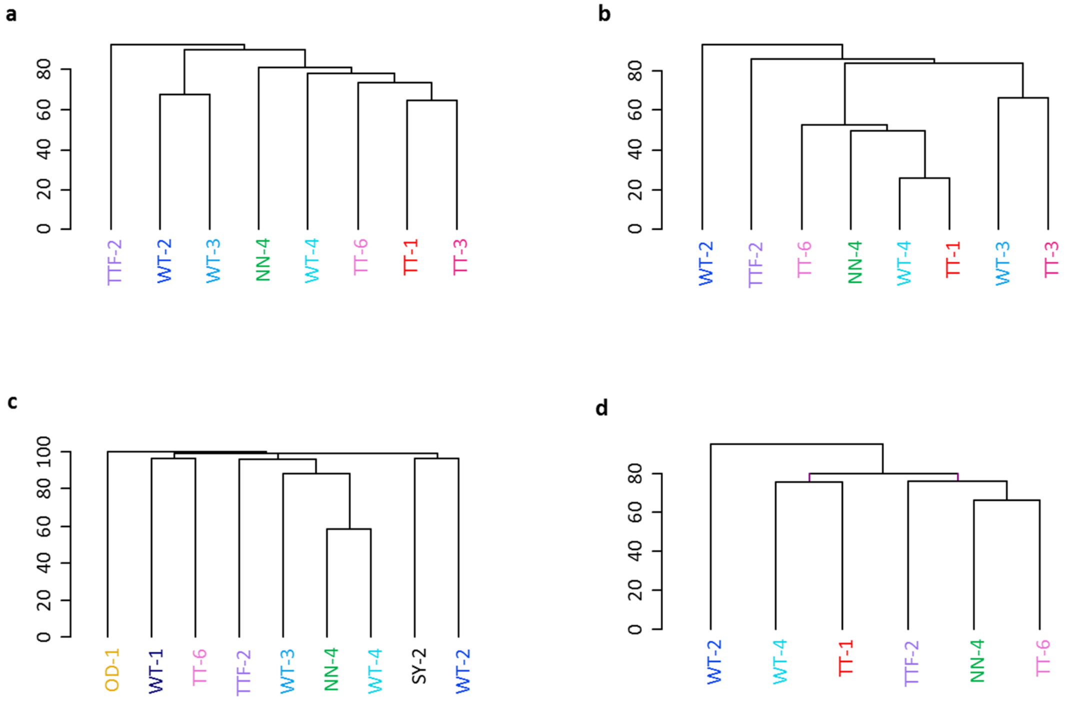

Figure S1. – Microbial community dissimilarities between the studied biocrust samples using SIMPROF analysis: a – bacteria, b – cyanobacteria, c – eukaryotes, d – fungi. SY, OD, WT, TT and NN correspond to Shreyahn, Oedesse, Wietze, Teutschenthal and not named site, respectively.

References

1. Nübel, U.; Garcia-Pichel, F.; Muyzer, G. PCR primers to amplify 16S rRNA genes from cyanobacteria. *Appl. Environ. Microbiol.* **1997**, *63*, 3327–3332.
2. Frey, B.; Rime, T.; Phillips, M.; Stierli, B.; Hajdas, I.; Widmer, F.; Hartmann, M. Microbial diversity in European alpine permafrost and active layers. *FEMS Microbiol. Ecol. Adv. Access* **2016**, *92*, fiw018, doi:10.1093/femsec/fiw018.
3. Stoeck, T.; Bass, D.; Nebel, M.; Christen, R.; Jones, M.D.M.; Breiner, H.W.; Richards, T.A. Multiple marker parallel tag environmental DNA sequencing reveals a highly complex eukaryotic community in marine anoxic water. *Mol. Ecol.* **2010**, *19*, 21–31, doi:10.1111/j.1365-294X.2009.04480.x.
